# P25 Healthcare associated candiduria in ICU patients: experience from a surveillance network in India, 2017–22

**DOI:** 10.1093/jacamr/dlad077.029

**Published:** 2023-08-02

**Authors:** Srividya K Vedachalam, Arpan Kumar Thakur, Rasna Parveen, Mamta Puraswani, Sharad Srivastav, Aparna Ningombam, Kamini Walia, Valan A Siromany, Daniel VanderEnde, Paul A Malpiedi, Amber M Vasquez, Purva Mathur, Indian Healthcare Associated Infection Surveillance Network Collaborators

**Affiliations:** India Epidemic Intelligence Service (EIS) Cell, National Centre for Disease Control (NCDC), New Delhi, India; Department of Lab Medicine, All India Institute of Medical Sciences (AIIMS), New Delhi, India; Department of Lab Medicine, All India Institute of Medical Sciences (AIIMS), New Delhi, India; Department of Lab Medicine, All India Institute of Medical Sciences (AIIMS), New Delhi, India; Department of Lab Medicine, All India Institute of Medical Sciences (AIIMS), New Delhi, India; Department of Lab Medicine, All India Institute of Medical Sciences (AIIMS), New Delhi, India; Indian Council of Medical Research, New Delhi, India; Division of Healthcare Quality Promotion (DHQP), US CDC, Atlanta, GA, USA; Division of Healthcare Quality Promotion (DHQP), US CDC, Atlanta, GA, USA; Division of Healthcare Quality Promotion (DHQP), US CDC, Atlanta, GA, USA; Division of Healthcare Quality Promotion (DHQP), US CDC, Atlanta, GA, USA; Department of Lab Medicine, All India Institute of Medical Sciences (AIIMS), New Delhi, India

## Abstract

**Background:**

Urinary tract infections (UTI) are common healthcare associated infections (HAI) in low-and-middle-income countries including India, with limited information on *Candida* UTI. An ICU-based HAI surveillance network of tertiary care hospitals across India, coordinated by the All-India Institute of Medical Sciences, New Delhi and the Indian Council of Medical Research, modified the US CDC’s National Healthcare Safety Network (NHSN) case definitions to record UTI. Unlike NHSN, India surveillance includes fungal pathogens, such as *Candida* spp., as eligible pathogens causing HAI-UTI. We describe the epidemiology, microbiology and antimicrobial resistance (AMR) profile of candiduria cases reported to this network to address the knowledge gap on *Candida* UTI in India.

**Methods:**

Patients with at least one UTI symptom and a positive urine culture with *Candida* sp. who had spent at least 2 calendar days in a participating ICU were included as candiduria events. All candiduria events reported by participating sites from 1 November 2017 to 31 May 2022 were extracted from overall UTI reported for all pathogens. The distribution of events by age, gender, ICU type, urinary catheterization, pathogen and drug resistance profile were performed. UTI rate and device utilization ratio (DUR) were calculated. Network sites performed antifungal susceptibility testing (AST) on *Candida* isolates using MIC-based method as per CLSI guidelines.

**Results:**

Data was reported by 131 ICUs in 39 hospitals. Overall network UTI events including all pathogens were 3600; UTI rates were 2.6 (adult ICUs) and 1.3 (paediatric ICUs); DUR was 0.8 (adult ICUs) and 0.4 (paediatric ICUs). There were 1111 candiduria events, 95% were classified as catheter-associated UTI (CAUTI), and the median age of patients was 65 years (IQR 32–74 years) and 58% were male. Among 739 *Candida* isolates speciated, *Candida tropicalis* (37%), *Candida albicans* (36%), *Candida glabrata* (6%) and *Candida auris* (6%) were the top four species isolated. Among 220 *C. tropicalis* isolates tested with AST, 6.9% were fluconazole resistant and 2% of 140 were caspofungin resistant; 7% of 177 *C. albicans* isolates were fluconazole resistant and 6.5% of 92 isolates were caspofungin resistant; and 48% of 28 *C. glabrata* isolates were fluconazole resistant and 44% of 18 isolates were caspofungin resistant.

**Conclusions:**

Candiduria events reported from ICUs to the HAI surveillance network-India are common and the majority are classified as CAUTI. Most of the species isolated are susceptible to fluconazole and caspofungin. Additional work is needed to understand the clinical significance of candiduria events reported to the network.

